# Paternal Involvement in Childcare and Housework and Mothers’ Spanking Behavior: The Japanese Longitudinal Survey of Newborns in the 21st Century

**DOI:** 10.2188/jea.JE20230270

**Published:** 2024-12-05

**Authors:** Mako Nagayoshi, Yuko Kachi, Tsuguhiko Kato, Manami Ochi, Yuichi Ichinose, Takayuki Kondo, Kenji Takehara

**Affiliations:** 1 Department of Preventive Medicine, Nagoya University Graduate School of Medicine, Nagoya, Japan; 2 Department of Social Medicine, National Center for Child Health and Development, Tokyo, Japan; 3 Department of Health Policy, National Center for Child Health and Development, Tokyo, Japan; 4 Department of Health and Welfare Services, National Institute of Public Health, Saitama, Japan; 5 Division of Health Services Research, Institute for Cancer Control, National Cancer Center, Tokyo, Japan; 6 Center for Research on Poverty among Children and Youth, Tokyo Metropolitan University, Tokyo, Japan

**Keywords:** childcare, housework, paternal involvement, spanking, Japan

## Abstract

**Background:**

No previous study reported an association of paternal involvement in childcare and housework with maternal physical punishment.

**Methods:**

Using data from the Japanese Longitudinal Survey of Newborns in the 21st century (*N* = 38,554), we analyzed responses about fathers’ involvement in childcare and housework at 6 months and mothers’ spanking of children at 3.5 years. Fathers’ involvement in childcare and housework was scored and categorized into quartiles. Spanking frequency was asked in the “often”, “sometimes”, or “not at all” categories. Multivariable-adjusted odds ratios (ORs) and 95% confidence intervals (CIs) for the mothers’ often spanking children were computed for the fathers’ involvement in childcare and housework. We also stratified the association by fathers’ working hours (40–49, 50–59, or ≥60 hours/week).

**Results:**

Among the 16,373 respondents, the proportion of mothers who often spanked their children was 4.8%. Compared with the lowest quartile, a higher frequency of paternal involvement in housework was associated with a lower risk of spanking children (*P_trend_* = 0.001). Adjustment for covariates attenuated the association, but significant association was observed in the 3^rd^ quartile of paternal involvement in housework (OR 0.77; 95% CI, 0.62–0.96). When the fathers worked fewer than 50 hours a week, a significant negative association was observed between the fathers’ frequency of childcare and the likeliness of the mothers’ spanking their children (*P_trend_* = 0.02).

**Conclusion:**

The fathers’ active involvement in childcare and housework could reduce the mothers’ physical punishment for their children.

## INTRODUCTION

Physical punishment of children is an act that intentionally causes physical pain or discomfort to children^1^ to control their words and actions. There is a growing consensus among health professionals that physical punishment of children is detrimental and ineffective. Currently, 65 countries prohibited physical punishment of children in all settings, and 27 other countries have committed to prohibiting it.^2^^,^^3^ In Japan, physical punishment by parents or other guardians was prohibited under the revised law on April 1, 2020.^4^ Spanking, a form of physical punishment,^1^ still occurs frequently worldwide.^3^ In the United States, the weighted frequency of spanking has been declining, but it is still reported from 37% of the parents with children aged 2–12 years.^5^ In Québec, Canada, 34.7% of the mothers or caregivers reported using physical punishment for their children aged 0–18 years in 2012 at least once per year. Cuartas et al^6^ estimated that in 2013, 62.5% (>220 million children) of the 2–4-year-old children living in 49 low- and middle-income countries were exposed to aggressive physical discipline (United Nations Children’s Fund’s Multiple Indicator Cluster Survey program). Baba et al^7^ reported the prevalence of spanking among Japanese parents in two national surveys, where the prevalence of “frequent” spanking was 9.9% in the first cohort, which started in 2001,^7^^,^^8^ and 5.2% in the second cohort, which started in 2010^8^; whereas, the prevalence of “sometimes” spanking was 67.3%, and 56.5%, respectively.^7^^,^^8^ Thus, spanking still occurs at a high frequency, and it is important to consider ways to prevent it.

Corporal punishment, despite not being severe, such as spanking,^2^ has long-term negative effects on the children’s growth and development and does not have positive outcomes.^2^^,^^9^ Many studies reported the serious risk of maternal spanking on child development and behavior.^10^^–^^12^ The altered brain response in children with lasting higher perception of threats induced by spanking was similar to the neural processing changes triggered by severe maltreatment, such as physical and sexual abuse.^12^

The exposure of mothers’ to childcare-related stress and environmental factors related to child rearing in Japan may be associated with spanking.^13^ Despite recent increases in the percentage of parental leave takers (17% in 2022 compared to 5% in 2017) and expanded options for paternal leave in workplaces,^14^ housework and caregiving continue to be predominantly perceived as women’s responsibilities in Japan, irrespective of their work status.^15^ The amount of time spent on childcare (49 min) and housework (34 min) per weekday by the Japanese fathers with children aged under 6 years was much less than that spent by the mothers (225 min and 229 min, respectively) in 2016.^16^ Although those durations among fathers have increased compared to 1996 (18 min and 20 min, respectively),^17^ they were still the least among the fathers in Organisation for Economic Co-operation and Development (OECD) countries.^18^ Especially, time spent on housework by Japanese fathers (34 min per weekday) was remarkably short; about one-fourth to one-third of other OECD countries.^18^ Longer working hours among Japanese men compared to OECD countries may contribute to this reason.^16^ Emerging Japanese terms explain the stressful situation for mothers; these terms include solo parenting or one-operator childcare (situation in which only the mother is responsible for childcare and housework),^19^ double shift (solo parenting with work outside home),^16^ or Ko-sodate (lonely childrearing without any support, including partner’s support). Maternal child-rearing stress and poor mental health status were recognized as strong risk factors for maternal physical punishment in children. Wilson et al^20^ reported that maternal parenting stress was associated with higher involvement of physical punishment in children aged up to three years in the United States. Niimura et al^21^ also reported that maternal parenting stress experienced at one and 36 months after birth predicted an approximately 70% higher involvement of physical punishment, even after adjusting for maternal depressive symptoms. Meanwhile, among 75,607 Japanese mothers, any involvement by fathers in childcare 6 months after delivery was associated with less maternal psychological distress 1 year after delivery.^22^ A study involving three ethnic groups in the United States^23^ revealed that fathers’ involvement in childcare duties had direct effects on lower parenting stress for the mothers and promoted maternal psychological adjustment after a 1-year follow-up period. However, there is lacking evidence on the association between paternal involvement in parenting and maternal corporal punishment, including spanking.

In this study, our aim was to elucidate the association of the frequency of fathers’ involvement in childcare and housework with the mothers’ use of physical punishment, specifically spanking children. We hypothesized that assessing this association might be challenging when fathers work long hours, as their absolute lack of time obscure their attitudes toward housework and childcare.^24^ Thus, we extended our analysis to include fathers who work shorter hours to clarify this relationship. By conducting a stratified analysis based on fathers’ working hours, we aimed to discern whether fathers would engage in housework and childcare if provided with more time.

## METHODS

### Design/setting and participants

The present longitudinal observational study is a secondary analysis of the Longitudinal Survey of Newborns in the 21st Century (LSN) in 2010, an ongoing annual household survey in Japan conducted by the Japanese Ministry of Health, Labour and Welfare. The target population of the LSN was all infants born in Japan between May 10 and 24, 2010 (*N* = 43,767) and their families. The main purpose of the LSN is for the central government to develop strategies against the low fertility trends in the Japanese society. Details of the LSN were previously described.^7^^,^^8^^,^^25^ We obtained permission to use this dataset from the Ministry of Health, Labour and Welfare of Japan. The study protocol was approved by the Ethics Committee of the National Center for Child Health and Development (No. 2020-299).

The first survey was conducted when the infants were 6 months old, and yearly follow-up surveys were conducted afterwards. We used the data from the first (6 months of age) and fourth (3.5 years of age) surveys. Both surveys utilized a self-administered questionnaire, and the responses were cross-referenced with birth record data to ascertain the child’s sex and gestational weeks. Information on the father’s and mother’s age was extracted from the child’s birth cohort data. The initial survey garnered responses from a total of 38,554 families.

**Figure 1.**
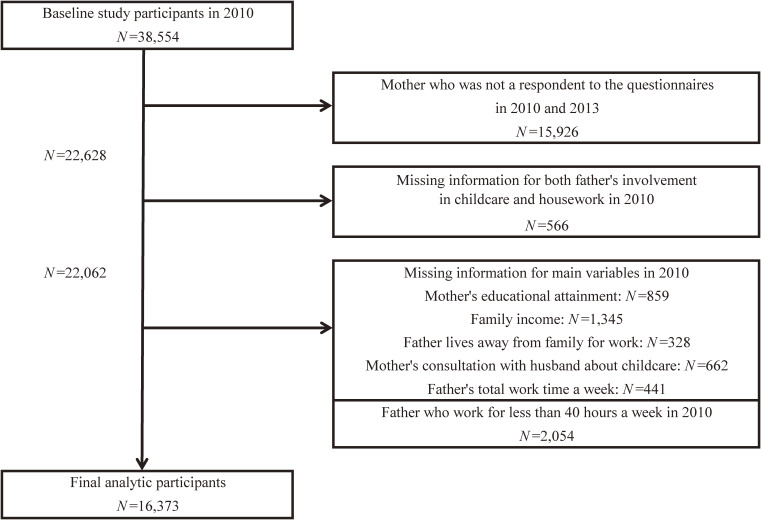
Study sample flow chart

To ensure a representative study sample, we implemented specific criteria, as illustrated in Figure 1. Recognizing variations in the distribution of the outcome (frequent spanking) and the exposure (fathers’ involvement in childcare and housework) among responders,^7^ we restricted responses to those provided by mothers. Notably, in the original dataset, fathers reported a higher frequency of involvement in childcare and housework compared to the reports from mothers. The percentage falling in the fourth quartile (the highest score group) was 1.4 times higher for childcare and 1.8 times higher for housework when reported by fathers. Additionally, we excluded fathers working shorter hours (less than 40 hours per week) due to the predominant representation of full-time workers (89%) with 40 or more hours per week in the target population. This exclusion aimed to mitigate potential confounding factors, such as health problems. Ultimately, the final sample comprised 16,373 families.

### Fathers’ involvement in childcare and housework

The frequency of paternal involvement in childcare and housework was reported by mothers during the first survey conducted at 6 months of age. Our choice to utilize fathers’ information at this specific time was driven by two main considerations. First, it aimed to eliminate the potential for reverse causation between the frequency of fathers’ childcare and housework and subsequent events, such as mothers’ spanking. By capturing fathers’ involvement at a time relatively distant from the survey for the outcome (mothers’ spanking), we sought to mitigate the influence of mothers’ spanking on fathers’ attitude (involvement in childcare and housework). Second, the postpartum is widely recognized as a critical phase for parents undergoing the significant life transition known as the “transition to parenthood”. Most couples experience sudden, small-to-medium deteriorations in relationship functioning after childbirth, persisting for at least 4 years.^26^^,^^27^ Additionally, the initial 6 months postpartum are crucial for the formation of mother and father-child attachments,^28^ as well as for children’s physical, mental, and social growth.^29^ Notably, the trajectory of fathers’ involvement revealed minimal dramatical changes during the 3 years.^30^

**Figure 2.**
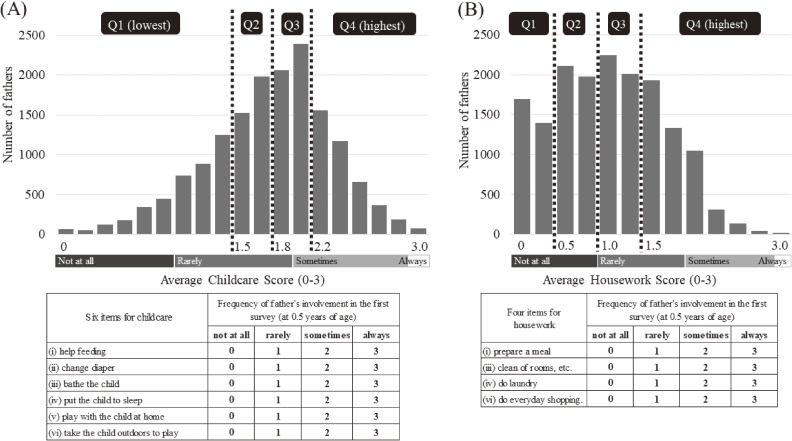
Distribution of paternal involvement in (**A**) childcare and (**B**) housework. The average score (standard deviation) of fathers’ involvement in childcare in 2010 (score range 0–3) was 1.7 (0.5), and the ranges of the quartile groups are 0–1.3, 1.5–1.7, 1.8–2.0, and 2.2–3.0, respectively. The average score (standard deviation) of fathers’ involvement in housework in 2010 (score range 0–3) was 1.0 (0.6), and the ranges of the quartile groups are 0–0.25, 0.5–0.75, 1.0–1.25, and 1.5–3.0, respectively. Q1, first quartile; Q2, second quartile; Q3, third quartile; Q4, fourth quartile.

Childcare duties included six items: (i) helping with feeding, (ii) changing diapers, (iii) bathing the child, (iv) putting the child to sleep, (v) playing with the child at home, and (vi) taking the child outdoors. Similarly, housework duties included six items; (i) preparing a meal, (ii) cleaning after meals, (iii) cleaning rooms, (iv) doing laundry, (v) taking out the trash, and (vi) doing everyday shopping. Of the 6-item housework, we excluded items (ii) and (v) because they were judged to be too light as a burden for housework. Large gaps between fathers and mothers were reported in the completion of procedures of their housework; fathers were not always aware of the entire process for these items.^31^^,^^32^ For example, husbands reported that they “take out the trash (45.1%)”, but they did not always include the preparation for that task (27.5%), such as gathering trash within the house.^31^ The corresponding proportions in wives were 75.1% and 95.9%.^31^ Similarly, 71% of husbands reported that they “carry the dishes after eating,” a part of eight processes of “(ii) cleaning up after meals.” However, their participation in the other seven processes, including “wash the dishes (44%),” were much lower than that (20–57%).^32^ Meanwhile, all eight processes were completed by around 90% of wives.^32^ The frequency of both childcare and housework was rated on a 4-point Likert Scale of: not at all (0), rarely (1), sometimes (2), and always (3). We summed the scores and divided by the number of total items. We then treated them as the average score per item for fathers’ childcare (ranged 0–3) and housework (ranged 0–3). Participants were categorized into quartiles for each childcare and housework score (Figure 2).

### Mothers’ spanking children

The frequency of spanking children by mothers was collected in 2013 (3.5 years of age) and was used as the outcome in the present study. When the behaviors of the children were not acceptable to the parents, the frequency of spanking children as a response was either “often,” “sometimes,” and “not at all.”

### Covariates

Covariates were assessed during the first and second survey. Information on the father’s and mother’s age (continuous), details on annual family income (continuous), living arrangements with grandparents (yes/no), consultation with husband about childcare (yes/no), and father’s working hours per week were collected during the first survey. Information on the father’s and mother’s educational attainment (high school graduate or less) was obtained in the second survey. Father’s working hours were asked with the following options: none, <20, 20–39, 40–49, 50–59, or ≥60 hours, and we excluded fathers who worked less than 40 hours per week. The number of siblings living with the family was also queried and categorized as no siblings, one, two, and three or more. Child’s sex (boy or girl) and gestational weeks were extracted from the birth record data. Cases with gestational weeks of 37 weeks or less were considered premature births.

### Statistical analyses

Descriptive statistics were generated for the mothers’ spanking children-related variables and covariates, including the fathers’ weekly working hours. Logistic regression models were used to estimate crude and adjusted odds ratios (ORs) and 95% confidence intervals (CIs) for the association of paternal involvement in childcare and housework with maternal spanking of children. ORs were computed for each category of childcare and housework (versus the first quartile [lowest frequency]). The adjusted model included mother’s age, child’s sex, mother’s educational attainment, annual family income, gestational weeks, number of siblings, living with grandparents, consulting with the husband about childcare, and fathers’ weekly working hours as covariates. We added a trend analysis to check for dose-response associations.

We also examined whether the fathers’ working hours per week modified the associations between the fathers’ involvement in childcare and housework and mothers’ spanking of children by including cross-product terms in the fully adjusted models. When an effect modification was observed, we further examined the association stratified by the fathers’ working hours per week (40–49, 50–59, or ≥60 hours).

All statistical analyses were performed using SAS (version 9.4; SAS Institute Inc., Cary, NC, USA). All *P*-values were two-tailed, and values <0.05 were considered statistically significant.

## RESULTS

Among the final analytic sample of 16,373 respondents, the proportions of mothers who “often” and “sometimes” spanked their children were 4.8% and 57.5%, respectively. The average score of the fathers’ involvement in childcare in 2010 (score range 0–3) was 1.7 (standard deviation [SD], 0.5), and each range of the respective four quartile groups was 0–1.3, 1.5–1.7, 1.8–2.0, and 2.2–3.0, respectively. The fathers’ involvement in housework in 2010 (score range 0–3) was 1.0 (SD, 0.6), and each range of the respective four quartile groups was 0–0.25, 0.5–0.75, 1.0–1.25, and 1.5–3.0, respectively. The distribution of fathers’ working hours per week was as follows: 36.8% worked 40–49 hours, 31.8% worked 50–59 hours, and 31.5% worked 60 hours or more.

**Figure 3.**
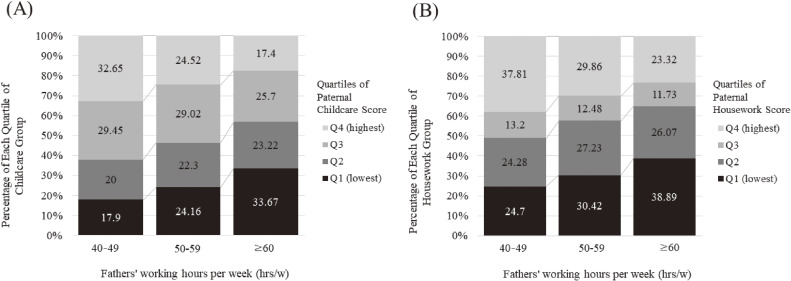
Distribution of quartiles of paternal involvement in (**A**) childcare and (**B**) housework by paternal working hours. The ranges of the quartile groups for paternal involvement in childcare in 2010 (score range, 0–3) are 0–1.3, 1.5–1.7, 1.8–2.0, and 2.2–3.0, respectively. The ranges of the quartile groups for paternal involvement in housework in 2010 (score range, 0–3) are 0–0.25, 0.5–0.75, 1.0–1.25, and 1.5–3.0, respectively. The proportions of fathers working hours per week (40–49 hours, 50–59 hours, and 60 hours or longer) are 36.8%, 31.8%, and 31.5%, respectively. Q1, first quartile; Q2, second quartile; Q3, third quartile; Q4, fourth quartile.

Table 1 shows the distribution of participants according to the fathers’ involvement in childcare and housework. The higher the fathers’ involvement in childcare, the higher the average score of their involvement in housework and vice versa. The distributions of mothers’ often spanking children according to paternal involvement in childcare and housework were not linear; however, the highest proportions were observed in the least involved groups (5.1% and 6.0%, respectively). The age of the mothers and fathers was younger according to the higher paternal involvement in childcare, but older according to higher paternal involvement in housework. The educational attainment of the mothers and fathers was higher with higher involvement in housework. Approximately half of the fathers who worked 40–49 hours per week (47.6% and 45.0%, respectively) were distributed among the most active involvement groups in childcare and housework (Table 1). Meanwhile, fathers who worked 60 hours or longer per week were highly distributed among the least involved groups in childcare and housework (42.4% and 41.9%, respectively) (Table 1). Within each group of fathers who worked 40–49, 50–59, and 60 hours or longer, the proportions of fathers who were least involved (scored 0 to 1.3: not at all to rarely) in childcare were 18.6%, 24.6%, and 34.3%, respectively (Figure 3); whereas, the proportions of fathers who were least involved in housework (scored 0 to 0.25: not at all) were 14.8%, 17.7%, and 25.3%, respectively (Figure 3).

**Table 1.** Socio-demographic characteristics of the study participants in 2010 by father’s involvement in childcare and housework score groups

	Childcare score quartiles				Housework score quartiles			
	
	Q1	Q2	Q3	Q4	Q1	Q2	Q3	Q4
	(least)			(most active)	(least)			(most active)
	*N* = 4,102	*N* = 3,519	*N* = 4,480	*N* = 4,018	*N* = 3,091	*N* = 4,094	*N* = 4,253	*N* = 4,824
**Father’s involvement in childcare in 2010 (average score of 6 items: 0–3)**								
Median (25–75%)	1.2 (0.8–1.3)	1.7 (1.5–1.7)	2.0 (1.8–2.0)	2.3 (2.2–2.5)	1.3 (1.0–1.7)	1.7 (1.3–2.0)	1.8 (1.5–2.2)	2.0 (1.8–2.3)
**Father’s involvement in housework in 2010 (average score of 4 items: 0–3)**								
Median (25–75%)	0.5 (0.0–1.0)	0.8 (0.5–1.3)	1.3 (0.8–1.5)	1.5 (1.0–1.8)	0.0 (0.0–0.3)	0.5 (0.5–0.8)	1.0 (1.0–1.3)	1.8 (1.5–2.0)

**Frequency of spanking child in 2013**								
Not at all, %	38.8	38.6	37.7	35.7	35.4	36.1	38.5	39.8
Sometimes, %	56.1	56.4	58.3	59.2	58.6	59.0	57.3	55.8
Often, %	5.1	5	4	5.1	6.0	4.8	4.3	4.4

**Age in 2010** ^a^								
Mother	32.2 (4.5)	31.9 (4.4)	31.8 (4.4)	31.5 (4.6)	31.5 (4.5)	31.8 (4.5)	32.0 (4.5)	32.1 (4.4)
Father	34.0 (5.2)	33.6 (5.1)	33.5 (5.2)	33.2 (5.4)	33.3 (5.2)	33.5 (5.2)	33.6 (5.2)	33.8 (5.3)
**Educational attainment: ≤high school graduate**								
Mother, %	29.4	25	25.6	30.3	32.6	28.8	28.5	22.8
Father, %	36.1	32.8	33.2	40	40.0	36.3	35.0	32.8

**Family income in 2010, million Japanese Yen**								
less than 200, %	1.6	1.4	1.2	1.5	1.8	1.5	1.2	1.1
200 to <400, %	20.0	16.2	16.7	19.9	21.3	19.1	18.9	15.3
400 to <600, %	34.7	34.3	36.8	38.4	37.6	36.7	36.4	34.3
600 to <800, %	22.7	24.2	23.8	22.3	21.0	23.4	23.5	24.4
800 to <1,000, %	11.2	13.2	12.2	10.7	10.2	11.0	11.0	14.1
≥1,000	9.8	10.7	9.3	7.2	8.1	8.3	9.0	10.8

**Child’s sex: boy, %**	49.9	50.4	52.2	52.1	52.0	50.2	52.6	50.2
**Weeks of gestation: <37 weeks, %**	4.1	4.6	4.8	6	3.8	5.0	4.5	5.9

**Number of family living with in 2010** ^a^	3.2 (1.3)	3.0 (1.2)	3.0 (1.2)	3.1 (1.3)	3.3 (1.3)	3.1 (1.3)	3.0 (1.2)	2.9 (1.1)
Living with grandparents, %	16.7	13.8	13.1	14.6	22.2	16.5	13.5	9.1
Number of siblings, %								
0	41.5	48.1	48.0	49.4	42.7	45.5	46.4	50.6
1	41.3	38.3	39.9	37.2	40.8	39.6	40.5	36.5
2	14.5	11.8	10.4	11.5	14.3	12.7	11.1	10.9
≥3	2.7	1.8	1.7	2.0	2.2	2.1	1.9	2.1

**Having a person who can consult with about childcare, %**	98.0	99.4	99.4	99.6	98.1	99.0	99.3	99.5
Consult with husband about childcare, %	76.0	88.8	92.7	94.0	77.2	86.7	90.5	93.5

**Mother’s total work time a week in 2010**								
>0 hours a week, %	10.7	8.6	8.9	13.1	12.0	9.5	9.4	10.7
**Father’s total work time a week in 2010**								
40–49 hours a week, %	26.9	33.8	38.4	47.6	28.6	31.9	37.9	45.0
50–59 hours a week, %	30.7	32.6	32.9	30.8	29.5	34.1	32.4	30.7
≥60 hours a week, %	42.4	33.6	28.8	21.6	41.9	34.0	29.7	24.2

*N*, number of participants; Q1, first quartile; Q2, second quartile; Q3, third quartile; Q4, fourth quartile.

^a^Mean (standard deviation).

### Fathers’ involvement in childcare and housework and mothers’ spanking children

#### Fathers’ involvement in housework

Compared with the lowest quartile, a higher frequency of paternal involvement in housework was associated with a lower risk of spanking children (*P_trend_* = 0.001) (Table 2). Adjustment for covariates attenuated the association, but the 3rd quartile of the higher frequency of paternal involvement in housework was still associated with a lower risk of mothers spanking children. The OR was 0.77 (95% CI, 0.62–0.96) (Table 2). There was no statistical evidence to suggest that the fathers’ working hours per week modified the relationship between fathers’ involvement in housework and often spanking of children (*P_interaction_* = 0.88).

**Table 2.** Adjusted association of father’s involvement in childcare in 2010 with mother’s spanking child often in 2013

		Number of spanking often	% of spanking often	Number at risk	Crude	Adjusted^*^
					OR (95% CI)	OR (95% CI)
**Father’s involvement in childcare in 2010**						
	Q1 (lowest)	211	5.1	4,102	Reference	Reference
	Q2	175	5.0	3,519	0.97 (0.79–1.19)	1.05 (0.85–1.30)
	Q3	178	4.0	4,480	**0.76 (0.62–0.94)**	0.82 (0.67–1.02)
	Q4 (highest)	205	5.1	4,018	0.99 (0.81–1.21)	1.03 (0.84–1.27)
	*P* for trend				0.42	0.70

**Mother’s age in 2010, years**		769	4.8	16,119		**0.96 (0.94–0.98)**

**Mother’s educational attainment:**						
	Above high school	479	4.1	11,667		Reference
	≤high school graduate	290	6.5	4,452		**1.32 (1.13–1.55)**

**Family income in 2010, per million Japanese Yen increase**		769	4.8	16,119		**0.94 (0.91–0.97)**

**Child’s sex**						
	Girl	269	3.4	7,869		Reference
	Boy	500	6.1	8,250		**1.82 (1.56–2.12)**

**Weeks of gestation**						
	≥37 weeks	728	4.7	15,332		Reference
	<37 weeks	41	5.2	787		1.10 (0.80–1.53)

**Number of siblings living with in 2010**						
	No siblings	340	4.5	7,528		Reference
	One	312	4.9	6,321		1.13 (0.96–1.33)
	Two	94	4.9	1,937		1.17 (0.91–1.49)
	Three	23	6.9	333		**1.65 (1.05–2.60)**

**Living with grandparents in 2010**						
	No	632	4.6	13,776		Reference
	Yes	137	5.8	2,343		1.10 (0.91–1.34)

**Having a person who can consult with about childcare**						
	No or Not husband	129	6.6	1,949		Reference
	Husband	640	4.5	14,170		**0.74 (0.61–0.91)**

**Father’s total work time a week in 2010**						
	40–49 hours a week	268	4.5	5,922		Reference
	50–59 hours a week	254	5.0	5,119		1.11 (0.93–1.33)
	≥60 hours a week	247	4.9	5,078		1.10 (0.92–1.32)

CI, confidence interval; OR, odds ratio; Q1, first quartile; Q2, second quartile; Q3, third quartile; Q4, fourth quartile.

^*^Adjusted for mother’s age (continuous) and child’s sex (girl/boy), mother’s education attainment (high school graduate or less), family income in 2010 (continuous), weeks of gestation less than 37 weeks (yes/no), number of siblings (0, 1, 2, and 3 or more), living with grandparents (yes/no), consult with husband about childcare (yes/no), father’s working hours in 2010 (40–49, 50–59, or ≥60 hours).

Results in **bold** indicate statistically significant at *P* < 0.05.

#### Fathers’ involvement in childcare

There was no overall association between the fathers’ involvement in childcare and spanking frequency (Table 3). However, there was statistical evidence that the fathers’ working hours per week modified the relationship between the fathers’ involvement in childcare and mothers’ frequent spanking of children (*P_interaction_* = 0.01). A clear trend was observed only among the fathers who worked 40–49 hours per week; the higher the fathers’ involvement in childcare, the less likely the mothers spanked their children (*P* = 0.02) (Table 4).

**Table 3.** Adjusted association of father’s involvement in housework in 2010 with mother’s spanking child often in 2013

		Number of spanking often	% of spanking often	Number at risk	Crude	Adjusted^*^
					OR (95% CI)	OR (95% CI)
**Father’s involvement in housework in 2010**						
	Q1 (lowest)	186	6.0	3,091	Reference	Reference
	Q2	198	4.8	4,094	**0.79 (0.65–0.98)**	0.86 (0.70–1.06)
	Q3	182	4.3	4,253	**0.70 (0.57–0.86)**	**0.77 (0.62–0.96)**
	Q4 (highest)	212	4.4	4,824	**0.72 (0.59–0.88)**	0.86 (0.69–1.06)
	*P* for trend				**0.001**	**0.12**

**Mother’s age in 2010, years**		769	4.8	16,119		**0.96 (0.94–0.98)**

**Mother’s educational attainment:**						
	Above high school	482	4.1	11,768		Reference
	≤high school graduate	296	6.6	4,494		**1.34 (1.14–1.57)**

**Family income in 2010, million Japanese Yen**		769	4.8	16,119		**0.94 (0.91–0.97)**

**Child’s sex**						
	Girl	273	3.4	7,938		Reference
	Boy	505	6.1	8,324		**1.81 (1.55–2.10)**

**Weeks of gestation**						
	≥37 weeks	871	5.0	17,383		Reference
	<37 weeks	42	4.6	909		1.08 (0.78–1.50)

**Number of siblings living with in 2010**						
	No siblings	343	4.5	7,598		Reference
	One	316	5.0	6,368		1.13 (0.96–1.33)
	Two	96	4.9	1,961		1.17 (0.92–1.49)
	Three	23	6.9	335		**1.63 (1.04–2.56)**

**Living with grandparents in 2010**						
	No	640	4.6	13,889		Reference
	Yes	138	5.8	2,373		1.07 (0.88–1.30)

**Person who can consult with about childcare**						
	No or Not husband	129	6.5	1,972		Reference
	Husband	649	4.5	14,290		**0.77 (0.63–0.95)**

**Father’s total work time a week in 2010**						
	40–49 hours a week	273	4.6	5,973		Reference
	50–59 hours a week	254	4.9	5,167		1.09 (0.91–1.30)
	≥60 hours a week	251	4.9	5,122		1.08 (0.91–1.29)

CI, confidence interval; OR, odds ratio; Q1, first quartile; Q2, second quartile; Q3, third quartile; Q4, fourth quartile.

^*^Adjusted for mother’s age (continuous) and child’s sex (girl/boy), mother’s education attainment (high school graduate or less), family income in 2010 (continuous), weeks of gestation less than 37 weeks (yes/no), number of siblings (0, 1, 2, and 3 or more), living with grandparents (yes/no), consult with husband about childcare (yes/no), father’s working hours in 2010 (40–49, 50–59, or ≥60 hours).

Results in **bold** indicate statistically significant at *P* < 0.05.

**Table 4.** Adjusted association of father’s involvement in childcare and housework in 2010 with mother’s spanking child often in 2013, stratified by father’s weekly work hours in 2010

Multivariate	Weekly work hours		

	40–49 hours	50–59 hours	≥60 hours
		
	OR (95% CI)^*^	OR (95% CI)^*^	OR (95% CI)^*^
**Father’s involvement in childcare in 2010**	*N* = 5,922	*N* = 5,119	*N* = 5,078
Q1 (lowest)	Reference	Reference	Reference
Q2	0.86 (0.59–1.24)	1.21 (0.83–1.76)	1.09 (0.77–1.55)
Q3	**0.64 (0.44–0.91)**	0.88 (0.60–1.29)	0.97 (0.68–1.39)
Q4 (highest)	**0.69 (0.48–0.97)**	1.38 (0.96–1.98)	1.20 (0.83–1.75)
*P* for trend	**0.02**	0.23	0.50

**Father’s involvement in housework in 2010**	*N* = 5,973	*N* = 5,167	*N* = 5,122
Q1 (lowest)	Reference	Reference	Reference
Q2	0.71 (0.48–1.04)	1.14 (0.77–1.67)	0.82 (0.59–1.16)
Q3	0.73 (0.51–1.05)	0.85 (0.56–1.29)	0.77 (0.54–1.09)
Q4 (highest)	0.71 (0.49–1.01)	1.24 (0.84–1.83)	0.72 (0.49–1.06)
*P* for trend	0.12	0.49	0.08

CI, confidence interval; *N*, number of participants; OR, odds ratio; Q1, first quartile; Q2, second quartile; Q3, third quartile; Q4, fourth quartile.

^*^Adjusted for mother’s age (continuous) and child’s sex (girl/boy), mother’s education attainment (high school graduate or less), family income in 2010 (continuous), weeks of gestation less than 37 weeks (yes/no), number of siblings (0, 1, 2, and 3 or more), living with grandparents (yes/no), consult with husband about childcare (yes/no).

Results in **bold** indicate a significant association with mothers’ frequent spanking (*P* < 0.05).

#### Remaining factors and mothers’ spanking children

The associations between each covariate and mothers’ frequent spanking are presented in Table 2 and Table 3. Mother’s lower educational attainment (high school graduate or less), having a male child, and having three or more siblings were positively associated with a higher risk of mothers’ frequently spanking. Older mother’s age, higher annual family income, and consultation with the husband about childcare (yes) were associated with a lower risk of mothers’ spanking their children often. Living with grandparents, gestational weeks, and fathers’ weekly working hours were not associated with mothers’ frequent spanking of children (Table 2 and Table 3).

## DISCUSSION

The fathers’ active involvement in childcare and housework could be related to a lower risk of mothers’ spanking of children among 16,373 mothers in Japan. Notably, the active involvement of fathers in childcare was associated with fewer events of spanking children by their mothers if the fathers worked less than 50 hours/week. To the best of our knowledge, this is the first study to assess the association between the fathers’ involvement in childcare and housework and mothers’ corporal punishment. This was assessed by “often” spanking children among a large national cohort study.

This study reveals that the involvement of fathers in housework and childcare may reduce spanking children by mothers. Some indirect pathways would be involved. Fathers’ involvement in housework and childcare may partially mediate an increase in the mothers’ physical and mental well-being and a decrease in maternal stress, which may partially prevent spanking by mothers. The previous studies showed that support deficits account for approximately 50% of higher stress among mothers.^33^ While, as previous study using this LSN data have shown that living with someone was associated with a lower risk of frequent spanking,^7^ family support from grandparents and other family members could be crucial. In addition, frequent paternal involvement in housework and childcare is a potential protective factor against maternal spanking. Fathers’ working hours did not influence the association for housework because housework can be done regardless of the fathers’ working hours and children’s bedtimes. The fathers’ involvement in housework, regardless of their working hours, may allow the mothers to spend quality time with their children by reducing their overall burden. Mothers who consult with their husbands about childcare had lower risk of spanking than who did not in our population (Table 2, Table 3); being with their partners could also provide a sense of security for the mothers.

This study revealed that a higher frequency of paternal childcare was associated with less spanking only when the paternal working hours were fewer than 50 hours/week. The difference in the association by fathers’ working hours may be partially explained by the different perception of the fathers’ attitudes by the mothers. For instance, when the fathers’ working hours are short and they are not involved in childcare at home, the mothers may perceive their involvement in childcare as less frequent than their expectations, which in turn could be associated with higher stress levels by the mothers. Conversely, when the fathers work for longer hours, there may be a sense of labor division, where the mothers take the lead in childcare. Mothers may have low expectations of the fathers’ involvement, as such fathers have less absolute time or cannot come home while their children are awake. Thus, the mothers’ stress could be relatively low and unlikely to contribute to the spanking behavior. A study in Japan reported that 70% of the Japanese men work for long hours, making it impossible for them to achieve the government’s target of 150 minutes per day for childcare and housework.^24^ However, the reason for the large gap in time spent on childcare and housework between fathers and mothers in Japan may not be fully explained by the long working hours of Japanese men.^18^ The robust gender role norms may also play a pivotal role in shaping domestic responsibilities in Japan; where a wide range in the fathers’ frequency of childcare was evident, even among the fathers who worked for relatively few hours (40–49 hours per week): about 18.6% of the fathers were in the least-involvement category (scored 0 to 1.3; not at all to rarely). Approximately 14.8% of the fathers who worked 40–49 hours per week were least involved in housework (scored 0 to 0.25; not at all). These show that gender gaps in domestic responsibilities are partially due to the evident gender role norms in Japan,^34^ which could be a stressful situation for mothers and influence their disciplinary behavior. Our results suggest that even in a society with strong gender role norms, the fathers’ active involvement in childcare when their working hours are relatively short is beneficial for preventing the mothers from spanking children.

As physical punishment has harmful influence on the children’s psychological development and behavior over time, efforts should be exerted to eliminate it. This study reports three potential findings. First, mothers need help and support with respect to childcare and daily housework duties. Second, paternal involvement helps mothers and may have benefits for creating a better environment for children. Third, direct support with childcare duties and indirect support, such as housework, influence the upbringing environment for children. **For the first finding,** the absolute amount of work performed by mothers was expected to be very high. This situation has not shown significant changes, despite the increase in the number of dual-earners.^16^^,^^35^ Childrearing requires a great deal of support, both inside and outside the home. However, with the current trend toward nuclear families and declining birthrates, childrearing support tends to be lacking and mothers are potentially more isolated. **For the second finding,** considering the influence of fathers on the mothers’ parenting attitudes, promoting appropriate working hours and norms in Japan is essential to provide support for mothers and fathers. Similar to previous reports,^24^ more than 60% of the fathers in this study worked very long hours, which prevented them from being involved in childrearing. Changing “norms” in the society and families in Japan is crucial because some fathers are least involved in childcare and housework even when they worked less than 50 hours per week as reported in this study. The “norms” include gender role norms^15^ and social and individual families’ recognition that fathers’ involvement in childcare and housework influences the mothers’ disciplinary behavior. In addition, the social norm that the parents should take responsibility for all the behaviors of their children needs to shift. People often say that “I’d like to see his (her) parents’ faces” when expressing shock at a child’s poor manners or behavior in Japan.^36^ Accordingly, the parents feel pressured and try to prevent their children from ill-behaving by spanking as a means of punishment. **For the third finding,** child-rearing environments should be improved to avoid the direct or indirect risk of high burden and isolation for mothers. In addition to the fathers’ involvement, changing social norms and maintaining mothers’ social connections, which could be achieved by having a job, especially full-time employment,^37^ may be crucial in reducing the risk of the mothers’ physical punishment. These factors are also important for detecting high-risk mothers in the early phase. More detailed longitudinal studies and intervention evaluations of the relationship between fathers’ involvement in childcare and housework and mothers’ physical punishment as well as factors that contribute to prevention (eg, working environment and norms in families and society) are needed in the future. This evidence will contribute to enhancing the environment for children. In the present study, we focused solely on evaluating the impact on mothers’ disciplining behavior, since mothers primarily played the key role in child-rearing at the time of the survey. However, even in scenarios where parental roles are shared or switched, and gender role norms become less biased, cooperation between the couples is expected to be necessary for creating a better environment for children. Further analysis should consider the evolving roles at home and explore additional dimensions of this complex dynamic.

This study had some limitations. First, a single question was used to assess the incidence of spanking children; thus, the detailed situations, such as the strength of spanking, could not be estimated. Due to differences in mothers’ perceptions of the behavior of spanking their children, possible underestimation due to misclassification may occur. For example, if mothers unconsciously spank their children, or if they perceive it as corporal punishment, they may respond with a lower frequency than they do. Second, as this self-reported measure was administered at a single point (at 3.5 years of age), the persistence of spanking (eg, temporal or long-lasting) was unknown. The severity of the situation varies according to the case. Third, the frequency of fathers’ involvement in childcare and housework at 6 months of age was based on the mothers’ responses. Fathers’ frequency of childcare and housework may reflect the mothers’ evaluations, satisfaction, and marital relationship; however, such information is not available. Fourth, our reliance on responses from mothers resulted in a final analysis with fewer subjects, less than half of the original population. However, this intentional restriction enabled precise stratification of participants using the same criteria. Previous research has indicated that the distribution of the mothers’ spanking frequencies and fathers’ involvement levels differs based on responders.^7^ Results calculated from mixed responders could introduce bias. For instance, the higher reported frequency of fathers’ involvement, as reported by fathers themselves, may be influenced by the actual characteristics of those fathers who cooperated with the survey. Additionally, this may be attributed to an evaluation bias, as fathers tend to provide higher self-ratings of their own housework and childcare frequency.^38^ Distinguishing these differences remains challenging. Finally, although we adjusted for potential confounders, unmeasured confounders (eg, mothers’ or fathers’ mental and physical conditions and children’s characteristics) could have influenced both fathers’ involvement in childcare and housework and mothers’ spanking. Despite these limitations, our study has several strengths. The data of the current study were obtained from nationally representative government statistical data (the LSN) with a large sample size, a wealth of information on potential confounding factors, and a longitudinal cohort design. This allowed for examining the association between the fathers’ involvement in childcare and housework six months after birth and subsequent mothers’ spanking at 3.5-years old, and whether they were dependent on the fathers’ working hours.

In conclusion, our results suggest that mothers are less likely to spank their children when their fathers engage in more childcare and housework. Although causal relationships should be confirmed by further studies with a longitudinal cohort design, paternal involvement may be beneficial in maintaining healthy disciplinary behavior by mothers. For a safer nurturing environment without physical punishment for children in Japan, it is crucial to explore the factors that influence the caregivers’ disciplinary behaviors (eg, fathers’ involvement in childcare and housework) and the contexts (eg, working hours and norms) that influence them.
